# Relationship Between Serum Complement C3 Levels and Outcomes Among Patients With Anti-GBM Disease

**DOI:** 10.3389/fimmu.2022.929155

**Published:** 2022-07-08

**Authors:** Mengyue Zhu, Jingjing Wang, Weibo Le, Feng Xu, Ying Jin, Chenfeng Jiao, Haitao Zhang

**Affiliations:** National Clinical Research Center of Kidney Diseases, Jinling Hospital, Medical School of Nanjing University, Nanjing, China

**Keywords:** anti-GBM disease, complement, C3, kidney failure, outcome

## Abstract

**Background:**

IgG and complement 3 (C3) are generally found to be deposited along the glomerular basement membrane (GBM) in human anti-GBM disease. The pathogenic role of complement activation in kidney damage of anti-GBM disease has been explored in recent years. Therefore, we investigated the relationship between serum C3 and outcomes among patients with anti-GBM disease in this study.

**Methods:**

Ninety-four anti-GBM disease patients between January 2004 and December 2020 at the National Clinical Research Center of Kidney Diseases Jinling Hospital were retrospectively analyzed, and were divided into the low C3 group and the normal C3 group according to serum C3 levels at diagnosis. Fifty-six patients had undergone renal biopsy. We analyzed the clinical manifestations, laboratory tests, kidney pathology, treatment, and outcomes between the two groups. The primary endpoint was kidney failure. Cox regression and smooth curve fitting of generalized additive mixed model analysis were used to explore the correlation between serum C3 and kidney failure. The outcomes of the two groups were compared by the Kaplan–Meier curve.

**Results:**

A total of 94 patients (aged 43.6 ± 16.2; male patients, 46%) with anti-GBM disease were enrolled. There were 26 patients with low C3 levels and 68 patients with normal C3 levels. Compared with the normal C3 group, patients in the low C3 group have a higher proportion of glomerular sclerosis progressing to kidney failure. Multivariate Cox regression analysis suggested that C3 is associated with kidney outcomes in patients with anti-GBM disease (HR = 0.782, 95% CI = 0.673–0.907, *p* = 0.001). Smooth curve fitting of generalized additive mixed model analysis indicated that the level of C3 had a linear relationship with the changing trend of kidney failure. The Kaplan–Meier curve showed that there was a statistical difference between the two groups in terms of kidney failure (*p* = 0.033).

**Conclusion:**

The kidney outcomes of anti-GBM disease in the low C3 group were poorer than those in the normal C3 group. The influence of C3 on the kidney outcomes of patients with anti-GBM disease may be of clinical relevance.

## Introduction

Anti-glomerular basement membrane (GBM) disease is a rare but life-threatening autoimmune disorder that is characterized by rapidly progressive glomerulonephritis with or without pulmonary hemorrhage. Kidney biopsy of anti-GBM disease has shown that immunoglobulin G (IgG) linearly deposits along the GBM, which is usually accompanied by linear or granular deposition of complement 3 (C3) (1). This indicates that complement activation may participate in the pathogenesis of anti-GBM disease. The pathways of complement activation in anti-GBM disease are mainly studied by passive injection of heterologous antibodies against GBM. It has been found that the complement system is activated in human anti-GBM disease through classical and alternative pathways (2), which plays an aggressive role in the pathogenesis of kidney injury by proinflammatory effect or cell lysis effect (3, 4). Multiple studies also showed that low serum C3 levels at diagnosis are associated with poor kidney outcomes in ANCA-associated vasculitis (5) and IgA nephropathy (6). However, to the best of our knowledge, few studies have analyzed the effect of serum C3 levels as a prognostic parameter in anti-GBM disease.

Therefore, this study aimed to investigate whether patients with low serum C3 levels at diagnosis have different clinical and histopathological features or outcomes compared to patients with normal C3 levels in anti-GBM disease.

## Materials and Methods

### Patients

**Figure 1:**
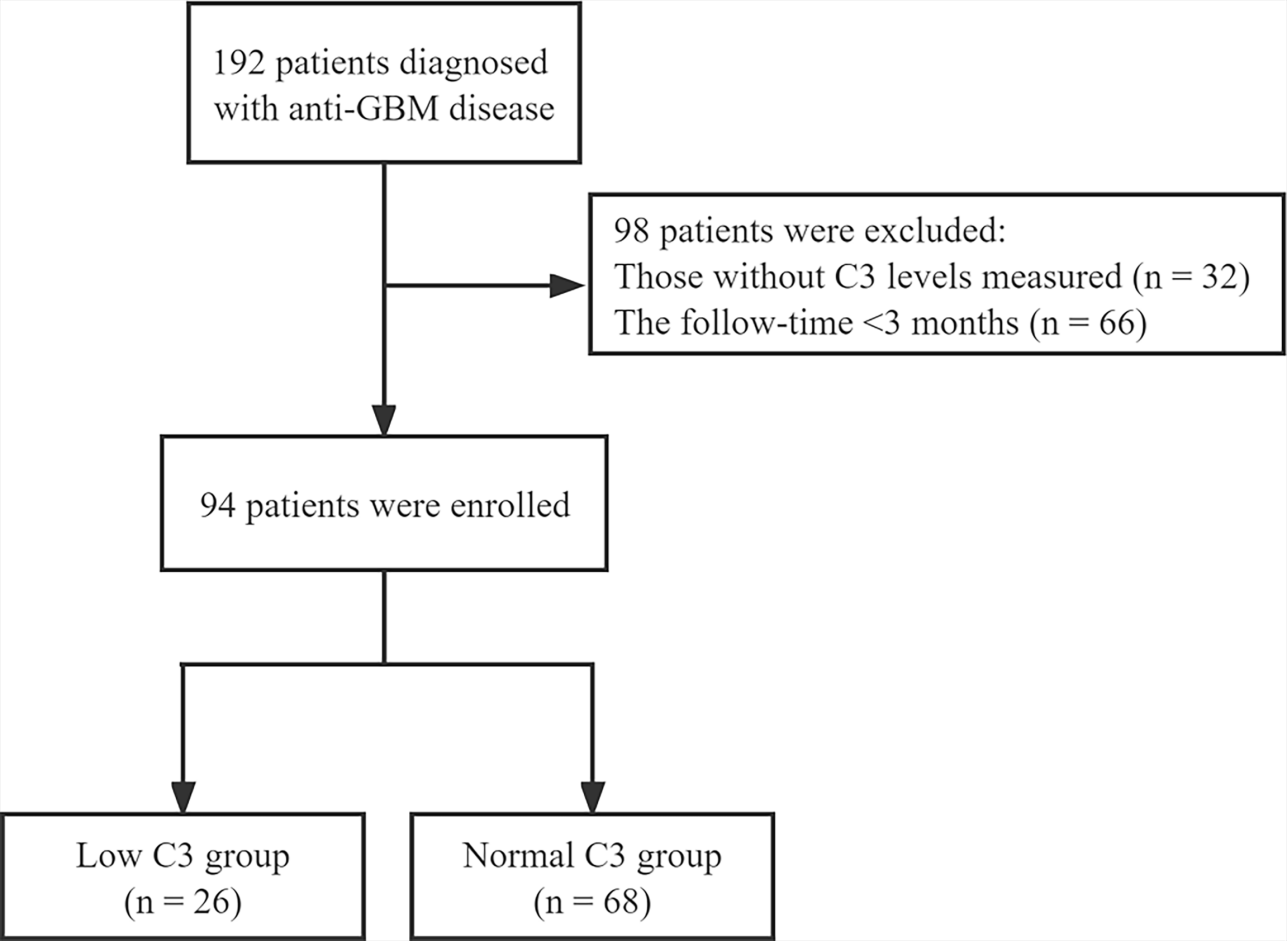
Study flowchart.

The patients diagnosed with anti-GBM disease in the National Clinical Research Center of Kidney Diseases Jinling Hospital from January 2004 to December 2020 were enrolled in this study. All patients had follow-up data for at least 3 months from time of presentation or until death, and those without serum C3 levels and missing follow-up records were excluded (**Figure 1**). The study complied with the Declaration of Helsinki and was approved by the independent ethics committee of Jinling Hospital (Approval 2022DZKY-033-01).

### Data Collection

Data of clinical, laboratory, and pathological variables were collected from medical records at diagnosis and during follow-up, which included the following: demographics, medical history and duration, clinical manifestations, serum C3 and C4, serum creatinine (SCr) at presentation, peak SCr, estimated glomerular filtration rate (eGFR), oliguria or anuria, hypertension, and kidney replacement therapy (KRT) at onset. Serum C3 and C4 levels were measured at the time of diagnosis, using a turbidimetric test. Our laboratory’s serum C3 reference range is 0.8–1.8 g/L, and serum C4 reference range is 0.1–0.4 g/L.

### Definitions

The main endpoint of the event was kidney failure, which was defined as the persistent need for KRT for more than 3 months. The need for KRT at onset was defined as the requirement for KRT during the first hospital stay. The eGFR was determined using the CKD-EPI equation (7). The extent of acute tubular–interstitial lesions includes tubular epithelial brush-border loss or interstitial edema and inflammatory cell infiltration area. Kidney tubular lesions used semi-quantitative scores: 0, not present; 1, present in 1%–25%; 2, present in 25%–50%; 3, present in >50%.

### Kidney Pathology

A kidney biopsy was performed at the time of diagnosis. Kidney specimens were evaluated by direct immunofluorescence and light and electron microscopy and were forwarded to two pathologists who examined the specimens separately, blinded to each other and the patients’ data.

For direct immunofluorescence, frozen sections were stained with a panel of fluorescein isothiocyanate-conjugated rabbit anti-human antibodies to IgG, IgM, IgA, C3, C1q, and fibrinogen. On light microscopy, the following indicators were evaluated by pathologists: the percentage of crescents (cellular, fibrocellular, and fibrous crescents), acute tubular–interstitial lesions, interstitial fibrosis, and tubular atrophy.

### Statistical Analysis

Values are expressed as mean ± standard (SD), or as median and interquartile range for continuous variables, and percentages for categorical variables. First, continuous variables were analyzed using the *t*-test. Meanwhile, the *χ*^2^ test and Mann–Whitney *U* test were used for categorical variables. Next, generalized additive model smooth curve fitting was used to address the relationship between serum C3 and kidney failure in anti-GBM disease, and univariate and multivariate Cox regression models were performed to examine whether C3 is associated with kidney failure in the disease. The Kaplan–Meier curve was used to analyze two groups’ kidney survival. In addition, an interaction test was conducted to evaluate whether patients’ characteristics influence the relationship between C3 and kidney failure. Data analysis was performed using R (http://www.r-project.org) and EmpowerStats (www.empowerstats.com.X&YSolutionsInc). A two-sided *p* < 0.05 was considered statistically significant.

## Results

### Patient Characteristics

A total of 94 patients diagnosed with anti-GBM disease were included in the study, 26 (27.7%) of whom had low C3 levels (below the normal range limit, <0.8 g/L). Patients were categorized according to the measurement of C3 into two groups, one group with low C3 and another group with normal C3. We found that percentage of glomerular sclerosis was higher in the low C3 group than the normal C3 group (*p* = 0.012), while there were no statistical differences in demographics, clinical, laboratory, and other pathological variables. The main clinical characteristics of the patients at presentation are summarized in **Table 1**. Among 94 patients, the average age at diagnosis was 43.6 ± 16.2, with 43 men and 51 women. The SCr at presentation was 661.9 ± 422.6 μmol/L, and the peak SCr was 896.1 ± 389.3 μmol/L. Mean serum C3 and C4 levels were 1.0 ± 0.3 and 0.2 ± 0.1 g/L, respectively. Kidney biopsies showed linear staining of IgG and linear or granular staining of C3 along GBM in the 36 (69%) patients. The average total crescent formation shown in the glomeruli was 68.5% (46.9%–88.8%) (**Table 2**). There were no statistical differences between the two groups of patients who received the standard induction therapy including glucocorticoids and cyclophosphamide combined with plasma purification technology. The median follow-up of the patients was 23.3 (7.1–74.0) months. At the last follow-up, 83 patients progressed into kidney failure, of which 26 were in the low C3 group and 57 (84%) were in the normal C3 group. There was no difference in patient survival between the low C3 group and the normal C3 group (*p* > 0.05).

**Table 1:** The baseline characteristics of patients with anti-GBM disease.

	Total (*N* = 94)	Low C3 group (*N* = 26)	Normal C3 group (*N* = 68)	*p*-value
Sex (male/female)	43/51	14/12	29/39	0.330
Age (years)	43.6 ± 16.2	44.4 ± 16.4	43.3 ± 16.2	0.773
Duration of disease (weeks)	4.5 (2.0, 10.8)	6.4 (3.0, 14.2)	3.7 (1.7, 8.6)	0.111
Exposure to chemicals, *n*	13 (14.3%)	3 (12.0%)	10 (15.2%)	0.701
Smoking history, *n*	16 (17.6%)	6 (24.0%)	10 (15.2%)	0.322
Oliguria/Anuria, *n*	51 (54.3%)	17 (65.4%)	34 (50.0%)	0.180
Pulmonary hemorrhage, *n*	18 (19.1%)	5 (19.2%)	13 (19.1%)	0.990
Hypertension, *n*	74 (78.7%)	23 (88.5%)	51 (75.0%)	0.154
RPGN, *n*	93 (98.9%)	26 (100.0%)	67 (98.5%)	0.534
KRT at onset, *n*	86 (91.5%)	24 (92.3%)	62 (91.2%)	0.860
Urinary protein (g/24 h)	1.6 (0.9, 3.2)	1.8 (1.0, 3.9)	1.5 (0.9, 3.2)	0.513
Microscopic hematuria (10^5^/ml)	1,075.0 (526.2, 2,592.5)	950.0 (320.0, 1,845.4)	1,250.0 (548.3, 3,000.0)	0.302
Hemoglobin (g/dl)	84.3 ± 17.0	80.0 ± 16.6	85.9 ± 17.0	0.135
Serum albumin (g/L)	31.8 ± 5.6	30.1 ± 5.9	32.5 ± 5.4	0.061
level of anti-GBM antibodies (RU/ml)	161.8 ± 75.6	136.5 ± 69.9	169.6 ± 76.1	0.088
eGFR (ml/min/1.73 m^2^)	8.1 (5.5, 11.7)	8.1 (4.9, 10.4)	8.1 (5.8, 13.6)	0.350
SCr at presentation (μmol/L)	661.9 ± 422.6	772.4 ± 592.3	619.6 ± 332.4	0.117
Peak SCr (μmol/L)	896.1 ± 389.3	987.5 ± 529.4	861.1 ± 318.3	0.160
C3 (g/L)	1.0 ± 0.3	0.7 ± 0.2	1.1 ± 0.2	<0.001*
C4 (g/L)	0.2 ± 0.1	0.2 ± 0.1	0.3 ± 0.1	<0.001*
Biopsy, *n*	56 (59.6%)	15 (57.7%)	41 (60.3%)	0.818
MP, *n*	79 (84.0%)	20 (76.9%)	59 (86.8%)	0.244
CTX, *n*	41 (43.6%)	11 (42.3%)	30 (44.1%)	0.874
Plasma purification, *n*	59 (62.8%)	16 (61.5%)	43 (63.2%)	0.879
PE, *n*	15 (16.0%)	8 (30.8%)	7 (10.3%)	0.015*
DFPP, *n*	9 (9.6%)	9 (34.6%)	30 (44.1%)	0.243
IA, *n*	39 (41.5%)	1 (3.8%)	8 (11.8%)	0.403
kidney failure, *n*	83 (88.3%)	26 (100.0%)	57 (83.8%)	0.029
Alive, *n*	90 (95.7%)	25 (96.2%)	65 (95.6%)	0.903
Follow-up period (months)	23.3 (7.1, 74.0)	23.3 (6.2, 47.5)	23.8 (7.3, 78.0)	0.386

RPGN, rapidly progressive glomerulonephritis; KRT, kidney replacement therapy; GBM, glomerular basement membrane; eGFR, estimated glomerular filtration rate; SCr, serum creatinine; MP, methylprednisolone; CTX, cyclophosphamide; PE, plasma exchange; DFPP, double filtration plasmapheresis; IA, immunoadsorption. *p < 0.05.

**Table 2:** The pathological characteristics of patients with anti-GBM disease.

	Total (*N* = 94)	Low C3 group (*N* = 26)	Normal C3 group (*N* = 68)	*p*-value
Biopsy, *n*	56 (59.6%)	15 (57.7%)	41 (60.3%)	0.818
Crescents (%)	68.5 (46.9, 88.8)	79.5 (56.8, 97.2)	66.7 (46.1, 84.9)	0.378
Cellular crescents (%)	19.6 (4.5, 41.3)	9.0 (0.0, 25.6)	27.2 (10.1, 44.7)	0.069
Fibrocellular crescents (%)	16.5 (0.8, 35.6)	19.2 (0.0, 55.7)	15.9 (4.6, 30.8)	0.803
Fibrous crescents (%)	0.0 (0.0, 12.3)	0.0 (0.0, 10.2)	0.0 (0.0, 11.8)	0.973
Glomerular sclerosis (%)	24.6 (5.0, 50.0)	50.0 (28.6, 65.2)	14.0 (2.7, 42.9)	0.004*
Acute interstitial lesions (scores)				0.725
0	12	4	8	
1	11	1	10	
2	9	3	6	
3	19	6	13	
Interstitial fibrosis and tubular atrophy (scores)				0.055
0	2	0	2	
1	15	2	13	
2	11	3	8	
3	23	9	14	
C3 deposition, *n*	36 (69.2%)	10 (71.4%)	26 (68.4%)	0.835
Granular deposition, *n*	27 (75.0%)	5 (50.0%)	22 (84.6%)	
Linear deposition, *n*	9 (25.0%)	4 (40.0%)	5 (19.2%)	

*p < 0.05.

### Predictors of Kidney Failure Among Patients With Anti-GBM Disease

Univariate analysis showed that C3 is markedly correlated with the risk of kidney failure. In addition, hypertension, oliguria or anuria, anti-GBM levels, eGFR, SCr at presentation, peak SCr, KRT at onset, crescents, and fibrocellular crescents were found to be associated with the risk of kidney failure (*p* < 0.05) (**Table 3**). After adjustment for age, gender, hypertension, anti-GBM levels, eGFR, KRT at onset, and crescents, multivariate analysis still showed that C3 at diagnosis was an independent protective factor for kidney outcomes of anti-GBM disease. For every 0.1 g/L increase in C3, the risk of developing kidney failure decreases by 22% (HR = 0.782, 95% CI = 0.673–0.907, *p* = 0.001) (**Table 3**). In addition, oliguria/anuria and peak SCr were also associated with kidney failure in multivariate analysis (**Table 3**).

**Table 3:** Predictors of kidney failure by univariate and multivariate Cox regression analysis.

Variable	Univariate analysis (*N* = 94)		Multivariate analysis (*N* = 94)	
	HR (95% CI)	*p*-value	HR (95% CI)	*p*-value
Age	1.008 (0.995–1.021)	0.243	1.004 (0.980–1.029)	0.736
Gender	1.051 (0.681–1.622)	0.822	1.321 (0.616–2.832)	0.474
Pulmonary hemorrhage	0.706 (0.394–1.266)	0.243	0.387 (0.138–1.087)	0.071
Hypertension	2.359 (1.309–4.249)	0.004*	1.794 (0.713–4.518)	0.215
Exposure to chemicals	0.900 (0.463–1.750)	0.756	0.627 (0.164–2.396)	0.495
Smoking	1.640 (0.938–2.870)	0.083	1.520 (0.516–4.479)	0.448
Oliguria/Anuria	4.468 (2.715–7.354)	<0.001*	7.764 (2.976–20.253)	<0.001*
Anti-GBM antibody levels (increased by 10 RU/ml)	1.045 (1.010–1.081)	0.012*	1.056 (0.993–1.123)	0.083
eGFR (ml/min/1.73 m^2^)	0.970 (0.944–0.997)	0.031*	0.992 (0.960–1.025)	0.612
SCr at presentation (increased by 88.4 μmol/L)	1.085 (1.042–1.130)	<0.001*	1.100 (0.958–1.264)	0.178
Peak SCr (increased by 88.4 μmol/L)	1.131 (1.086–1.177)	<0.001*	1.199 (1.039–1.384)	0.013*
Hb (increased by 10 g/L)	0.950 (0.843–1.070)	0.395	1.141 (0.878–1.482)	0.324
C3 (increased by 0.1 g/L)	0.867 (0.798–0.941)	<0.001*	0.782 (0.673–0.907)	0.001*
C4 (increased by 0.1 g/L)	0.819 (0.666–1.006)	0.056	0.708 (0.484–1.035)	0.075
KRT at onset	11.671 (2.811–48.447)	<0.001*	4.748 (0.511–44.141)	0.171
C3 deposition	1.069 (0.564–2.024)	0.839	0.732 (0.340–1.578)	0.426
Crescents (increased by 10%)	1.137 (1.012–1.278)	0.031*	1.087 (0.941–1.255)	0.256
Cellular crescents (increased by 10%)	0.988 (0.879–1.109)	0.833	0.972 (0.827–1.141)	0.726
Fibrocellular crescents (increased by 10%)	1.412 (1.012–1.288)	0.031*	1.179 (1.000–1.389)	0.050
Fibrous crescents (increased by 10%)	1.064 (0.918–1.233)	0.411	1.179 (1.000–1.389)	0.119
Glomerular sclerosis (increased by 10%)	1.118 (0.999–1.251)	0.053	1.059 (0.919–1.219)	0.429

GBM, glomerular basement membrane; eGFR, estimated glomerular filtration rate; SCr, serum creatinine; Hb, hemoglobin; KRT, kidney replacement therapy; Hazard ratio (HR) of multivariate analysis was adjusted for age, gender, hypertension, anti-GBM levels, eGFR, KRT at onset, and crescents. *p < 0.05.

### Association Between Serum C3 and Outcomes of Anti-GBM Disease

**Figure 2:**
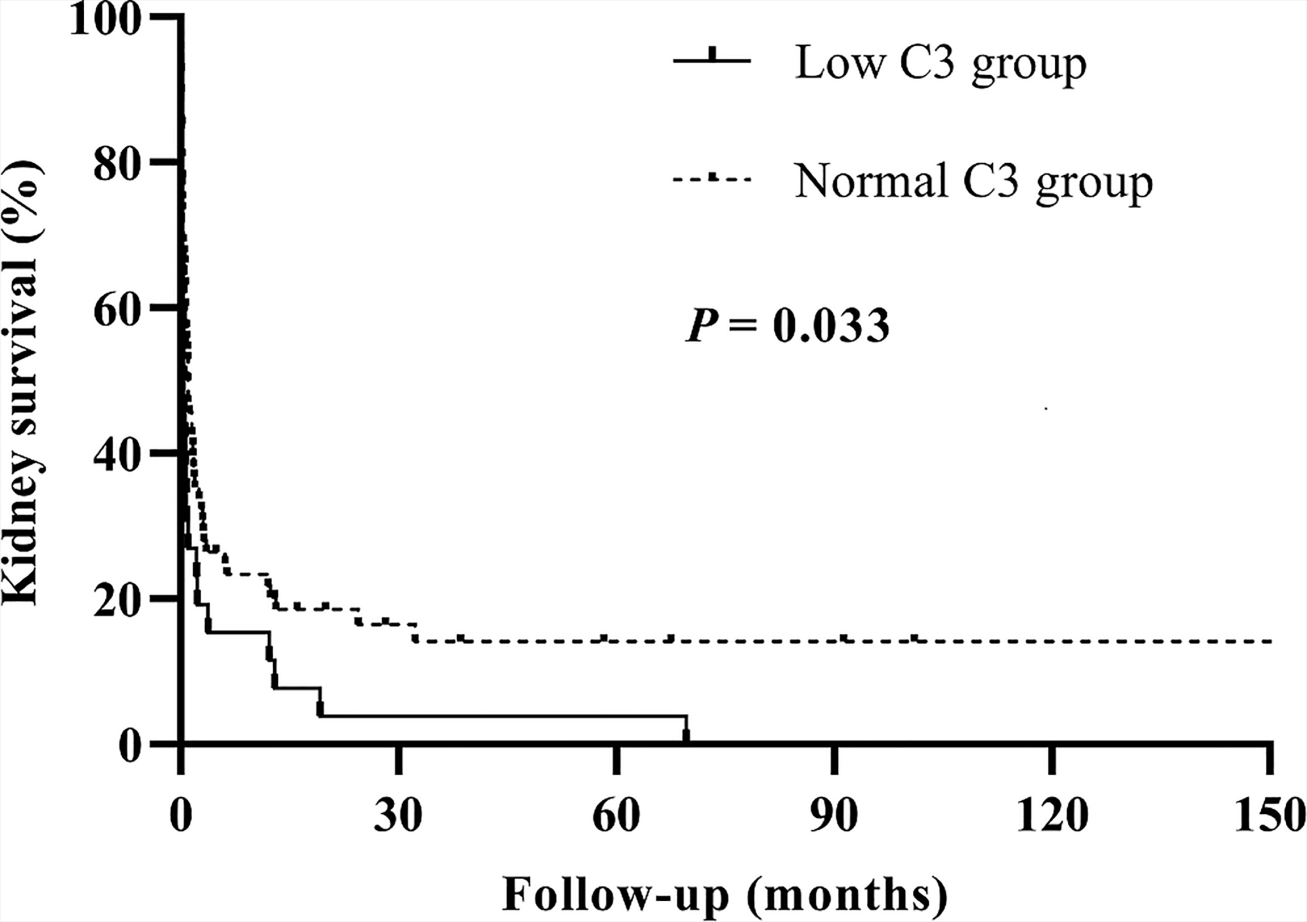
Kaplan–Meier survival analysis for kidney outcomes between the low C3 group and the normal C3 group.

We analyzed the patient and kidney survival according to C3 levels, finding that patients in the low C3 group had significantly worse kidney survival than the normal C3 group (*p* = 0.029). We further compared kidney survival of patients with anti-GBM disease in two groups by the Kaplan–Meier curve, which showed that low C3 was associated with a significantly lower kidney survival (*p* = 0.033) (**Figure 2**).

**Figure 3:**
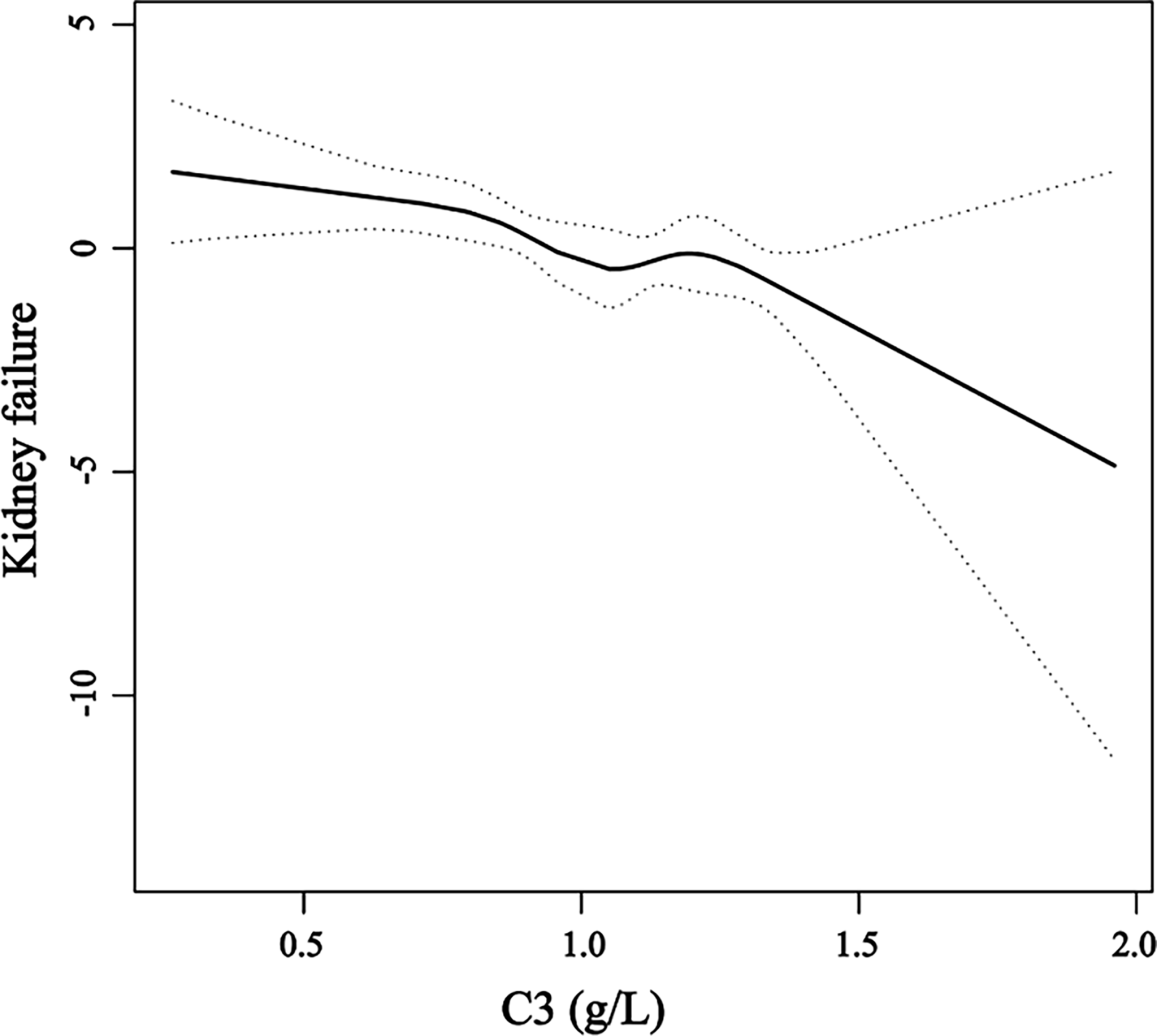
A smooth curve fitting of the relationship between serum C3 levels and the risk of kidney failure. The black straight line represents the smooth curve fit between the variables. The dotted line represents the 95% confidence interval of the fit.

Smooth curve fitting of C3 and kidney failure was conducted after adjustments for age, gender, hypertension, anti-GBM levels, eGFR, KRT at onset, and crescents. The results showed that C3 level was linearly correlated with the changing trend of kidney failure risk (*p* = 0.001), which means that as C3 levels increase, the risk of kidney failure occurrence gradually decreases (**Figure 3**).

In this study, the relationship between C3 and kidney failure was further stratified by age, hemoglobin, serum albumin, SCr at presentation, oliguria or anuria, and crescents. The results suggested that when the serum albumin <30 g/L, C3 had a protective effect on outcomes in patients with anti-GBM disease, and serum albumin could modify the relationship between C3 and kidney failure. In addition, the protective role in outcomes of C3 was observed only in patients without oliguria/anuria (HR = 0.029, 95% CI = 0.002–0.335, *p* = 0.005). Serum albumin levels and oliguria/anuria were considered the prominent interactive factors that affect the association between C3 and the risk of kidney failure by the interaction analysis, which remained robust under the grouping of other indicators (**Table 4**).

**Table 4:** Relationship between C3 and kidney failure under different stratification factors.

Subgroup stratification	Cases	HR (95% CI)	*p*-value	*p* for interaction
Age (years)				0.652
<60	75	0.076 (0.015–0.379)	0.002	
≥60	19	0.062 (0.001–5.497)	0.224	
Hemoglobin (g/L)				0.106
<90	62	0.034 (0.006–0.187)	<0.001	
≥90	32	0.466 (0.031–7.065)	0.582	
Serum albumin (g/L)				0.040*
<30	40	0.011 (0.001–0.123)	<0.001	
≥30	54	0.189 (0.033–1.082)	0.061	
SCr at presentation (μmol/L)				0.760
<500	31	0.110 (0.014–0.878)	0.037	
≥500	63	0.074 (0.010–0.535)	0.010	
Oliguria/anuria				0.024*
No	43	0.029 (0.002–0.335)	0.005	
Yes	51	0.725 (0.096–5.461)	0.755	
Crescents (%)				0.265
<60	21	0.024 (0.002–0.377)	0.008	
≥60	33	0.127 (0.026–0.610)	0.010	
C3 deposition				0.888
No	16	0.094 (0.005–1.661)	0.107	
Yes	36	0.075 (0.015–0.378)	0.002	

Hazard ratio (HR) was adjusted for age, gender, hypertension, anti-GBM levels, eGFR, KRT at onset, and crescents. *p < 0.05.

## Discussion

This is the first retrospective study to explore the relationship between serum C3 levels measured at diagnosis and the risk of kidney failure in patients with anti-GBM disease. We observed that patients with low C3 levels had more severe glomerular sclerosis and poorer kidney survival than normal C3 levels. Serum C3 may be a protective factor for kidney outcomes. Furthermore, we confirmed that C3 was associated with kidney outcomes by univariable and multivariable Cox regression. Meanwhile, there was a difference in the Kaplan–Meier curve between the low C3 and the normal C3 groups. Therefore, we thought that serum C3 at diagnosis has a protective effect on kidney prognosis in patients with anti-GBM disease. In addition, our work suggested that serum albumin and oliguria/anuria were considered the prominent interactive factors that affect the relationship between C3 and the risk of kidney failure by the interaction analysis.

The complement system plays an essential role in immune-mediated glomerulonephritis. C3 deposition of the glomerular capillary wall can be shown by immunofluorescence, which is consistent with the assumption that complement activation participated in the generation of kidney damage of human anti-GBM disease. Previous studies of humans and animals both have found that the complement system is activated through the classical and alternative pathways in anti-GBM disease (2, 8). Some researchers supported the idea that antibody-directed complement activation initiates a cascade involving cytokine production, upregulation of adhesion molecules and chemokine production, and recruitment and activation of platelets and neutrophils, which ultimately lead to this early glomerular injury (9–12). Ma et al. have found that the complement cascade played a pathogenic role in kidney injury, as shown by the possible proinflammatory effect of C5a and/or cell lysis effect of C5b-9 in 20 patients with kidney biopsy-confirmed anti-GBM disease (8).

Several animal models have been employed to better understand the complement system’s role in the development of anti-GBM disease. Hammer et al. discovered that the primary stage of nephrotoxic serum nephritis produced by rabbit nephrotoxic serum appears to a great extent, but not wholly, upon the participation of serum complement (13). Sheerin et al., using C3-deficient mice, found that complement acts synergistically with heterologous antibodies, resulting in neutrophil infiltration and glomerular injury in experimental anti-GBM disease. Their studies also supported that classical and alternative pathways of the complement system are involved in the development of anti-GBM disease (14). Moreover, in the different phases of anti-GBM disease, the role of complement is contradictory. They thought that the effect of complement in the pathogenesis of glomerular disease might be dependent on the stage of the disease (15). More importantly, they also compared the contribution of systemic and local production of C3, concluding that circulating C3 is a critical factor in reducing the glomerular accumulation of immune complexes, while local synthesis of C3 did not have a major influence on this aspect of glomerular disease (16).

Our study found that lower C3 levels are associated with poorer kidney survival. There were no statistical differences between the two groups of patients who received the standard induction therapy including glucocorticoids and cyclophosphamide combined with a plasma purification technique. Even after adjusting for age, gender, hypertension, GBM, eGFR, KRT at onset, and crescents, C3 levels remained significantly associated with kidney survival. We further found that the C3 level was linearly related to the changing trend of kidney failure, and the risk of kidney failure gradually decreased as the C3 levels increased, which would suggest that serum C3 levels at diagnosis of anti-GBM disease could be an independent protective factor, but this needs further confirmation.

In addition, we found that serum albumin level was considered the prominent interactive factor that affects the association between C3 and the risk of kidney failure by the interaction analysis, which may be related to complement involvement in the pathogenesis of proteinuria (17). Considering that both albumin and C3 may be related to the nutritional status of patients and are potential systemic inflammatory response proteins, there may be other factors at play resulting in a concurrent decrease in both albumin and C3 (and maybe C4), which, in turn, results in worse outcomes that need to be further explored. As for C3, there was no protective role in patients with oliguria or anuria, which may be explained by oliguria or anuria being a stronger predictor of kidney survival in patients with anti-GBM disease compared with other variables, and almost all patients with initial oliguria or anuria end up entering kidney failure (18). Serum C4 levels and local synthesis of C3 have not been found to be associated with kidney outcomes of the disease in our study. We discovered that oliguria/anuria and peak SCr were risk factors for kidney failure in multivariable regression analysis, similar to previous reports.

Although C3 has been rarely studied in human anti-GBM disease, the crucial role of complement in pathogenesis has been explored in IgA nephropathy (6), C3 glomerulonephritis (19), membranous nephropathy (20), and other nephritis (21). Many recent studies demonstrated that C3 levels are correlated to patient and kidney survival in ANCA-associated vasculitis as well (22–24). The critical role of C3 at the intersection of all complement activation pathways and its synergistic effect in multiple immune and inflammatory networks have facilitated the development of C3-based therapies (25). Now, C3 intervention is emerging as a viable therapeutic strategy for rare inflammatory kidney diseases, such as C3 glomerulopathy (26).

Nevertheless, this study still has some limitations, including the small sample size and the single-center design. Moreover, some patients did not have a kidney biopsy at diagnosis, thus limiting the histological analysis. Despite these limitations, we demonstrated clearly that serum C3 plays a protective role in the progress of anti-GBM disease and that low serum C3 level at diagnosis is associated with poor kidney outcomes.

In conclusion, this study retrospectively compared the clinicopathological features and outcomes between the low C3 group and the normal C3 group, and found that patients with low serum C3 levels had a higher proportion of glomerular sclerosis progressing into kidney failure. Serum C3 is closely related to the kidney outcomes of patients with anti-GBM disease, and the pathogenesis of C3 in the disease needs to be further studied.

## Data Availability Statement

The original contributions presented in the study are included in the article/supplementary material. Further inquiries can be directed to the corresponding author.

## Ethics Statement

The studies involving human participants were reviewed and approved by Independent ethics committee of Jinling Hospital. Written informed consent from the participants’ legal guardian/next of kin was not required to participate in this study in accordance with the national legislation and the institutional requirements.

## Author Contributions

JW and HZ contributed to the conception of the study. WL screened the data, and MZ analyzed and interpreted the data. FX completed the pathological analysis. MZ, JW, YJ, and CJ contributed to the follow-up. MZ finished the manuscript. JW and HZ supervised and edited the manuscript. All authors contributed to the work and approved the submitted version for publication.

## Funding

This work was supported by the National Key Research and Development Project of China (2021YFC2501302) and the Jiangsu Clinical Research Center Project (YXZXA2016003).

## Conflict of Interest

The authors declare that the research was conducted in the absence of any commercial or financial relationships that could be construed as a potential conflict of interest.

## Publisher’s Note

All claims expressed in this article are solely those of the authors and do not necessarily represent those of their affiliated organizations, or those of the publisher, the editors and the reviewers. Any product that may be evaluated in this article, or claim that may be made by its manufacturer, is not guaranteed or endorsed by the publisher.
